# Late-Onset Arrhythmogenic Right Ventricular Cardiomyopathy Mimicking Acute Coronary Syndrome in an Elderly Female: A Case Report

**DOI:** 10.7759/cureus.96088

**Published:** 2025-11-04

**Authors:** Aung Hein, Ei M Mon, Ma. Clarissa Solomon, Myo M Htet

**Affiliations:** 1 Department of Cardiology, University Hospitals Dorset, Bournemouth, GBR; 2 Department of Internal Medicine, Princess Alexandra Hospital, Brisbane, AUS

**Keywords:** acute coronary syndrome (acs), arrhythmogenic right ventricular cardiomyopathy (arvc), myocardial fibrofatty replacement, pkp2 gene mutation, ventricular tachycardia

## Abstract

Arrhythmogenic right ventricular cardiomyopathy (ARVC) is a cardiac condition characterized by the replacement of myocardial tissue with fibrofatty tissue, primarily affecting the right ventricle. Though typically presenting in younger individuals, this case report discusses a rare presentation in a 72-year-old female who exhibited symptoms mimicking acute coronary syndrome (ACS), including chest pain and sustained ventricular tachycardia (VT). Coronary angiography revealed normal coronary anatomy, ruling out ACS as the underlying cause. Cardiac MRI demonstrated structural abnormalities in the right ventricle, consistent with ARVC. Genetic testing confirmed a pathogenic mutation in the PKP2 gene. The patient was treated with antiarrhythmic medications and received an implantable cardioverter-defibrillator (ICD) for secondary prevention of sudden cardiac death. This report emphasizes the importance of considering ARVC in the differential diagnosis of elderly patients with unexplained ventricular arrhythmias and chest pain, particularly when coronary angiographic findings are normal.

## Introduction

Arrhythmogenic right ventricular cardiomyopathy (ARVC) is a cardiac condition characterized by the progressive replacement of myocardial tissue with fibrofatty tissue, primarily affecting the right ventricle [1]. Although originally believed to affect only the right ventricle, ARVC is now recognized to also involve the left ventricle or manifest as a biventricular disorder [2]. Typically inherited in an autosomal dominant pattern, the penetrance and gene expression of ARVC vary significantly among individuals [3,4]. In the general population, the prevalence of ARVC is estimated to be approximately 1:2,000 to 1:5000, with higher rates reported in specific regions such as the island of Naxos in Greece and parts of Italy, where prevalence reaches 0.4-0.8% [5].

ARVC is primarily caused by mutations in desmosomal genes, which are crucial for maintaining the structural integrity of cardiac muscle. These mutations lead to progressive deterioration of myocardial tissue, ultimately resulting in scar formation and fatty infiltration. The most commonly implicated genes include desmoplakin (DSP), desmoglein 2 (DSG2), desmocollin 2 (DSC2), plakoglobin (JUP), and plakophilin 2 (PKP2) [1]. PKP2 is the predominant gene associated with ARVC, and in one study of 120 individuals, roughly one-quarter (26%) carried a PKP2 mutation [1]. When the disease involves the left ventricle or both ventricles, non-desmosomal gene mutations are more frequently observed, further complicating diagnosis and management.

The clinical presentation of ARVC is heterogeneous, with common symptoms including palpitations and syncope, particularly during physical activity. Some patients may experience dyspnoea, peripheral oedema, or signs of right heart failure, while sudden cardiac death may be the first manifestation in others. Additionally, ECG changes and elevated cardiac enzymes that mimic acute coronary syndrome (ACS) can occur, as observed in this case [6].

Diagnosing ARVC in elderly patients poses unique challenges, as the disease typically manifests in younger individuals. However, a study of 502 ARVC patients revealed that 21% were diagnosed at age 50 or older, with sustained ventricular tachycardia (VT) being the predominant manifestation [7]. Patients with late presentation are less likely to exhibit precordial T-wave repolarization abnormalities or ventricular ectopy. Late presentation of ARVC has been associated with factors such as male sex, absence of family history, genetic mutations, right ventricular structural abnormalities, and inducible VT on electrophysiology studies, all of which confer an increased arrhythmic risk in this population. Our case exhibited three of these features: right ventricular structural disease, a pathogenic variant, and no known family history of ARVC. Consequently, definitive diagnosis in elderly patients can be challenging, often resulting in initial misdiagnosis of conditions such as ACS.

## Case presentation

**Figure 1:**
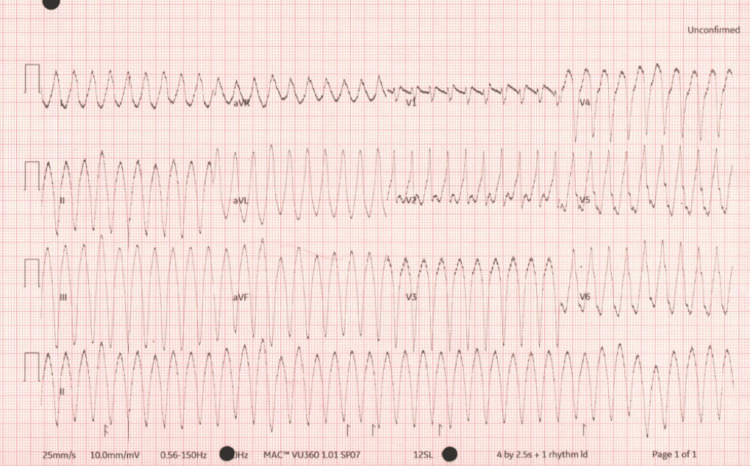
First ECG upon presentation ECG demonstrating broad complex tachycardia with pulse (pulsed VT) ECG: electrocardiogram; VT: ventricular tachycardia

A 72-year-old woman presented after experiencing chest tightness and dizziness while participating in a Zumba class. She also reported cold, clammy extremities, four episodes of vomiting, shortness of breath, and transient visual changes. Her past medical history included well-controlled hypertension managed with a single antihypertensive medication. There was no family history of sudden cardiac death. When assessed by paramedics, the electrocardiogram (ECG) demonstrated VT, and she had maintained pulse. Upon arrival at the emergency department, she remained in sustained VT with left bundle branch block (LBBB) morphology (Figure 1).

**Figure 2:**
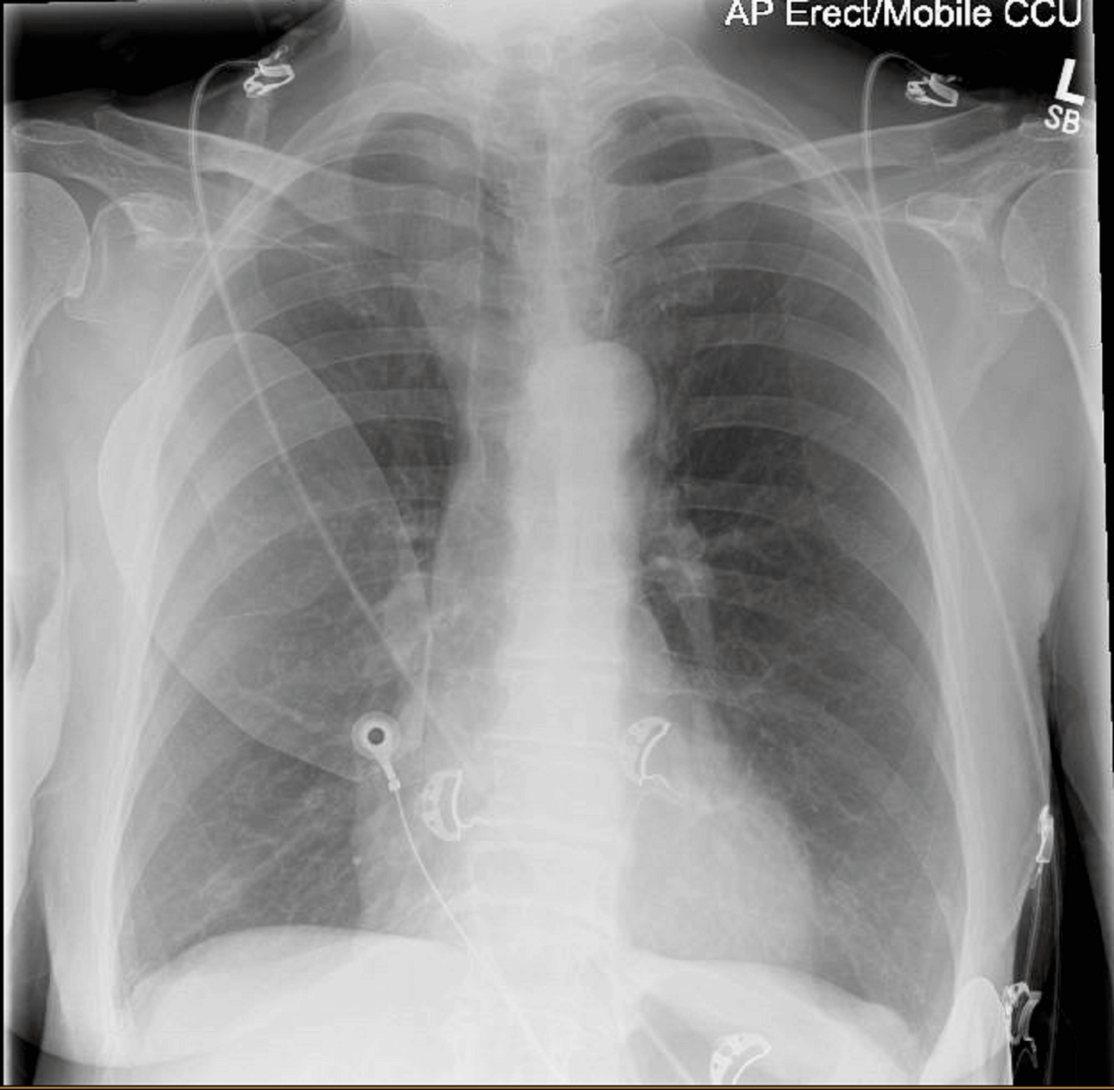
Chest radiograph Chest X-ray did not show any pulmonary edema

On examination, her Glasgow Coma Scale (GCS) score was 15/15, heart rate was 235 bpm, and blood pressure was 58/36 mmHg. She showed no signs of fluid overload. Initial venous blood gas analysis revealed mild hyponatremia (sodium 128 mmol/L), metabolic acidosis (pH 7.267, bicarbonate 17.9 mmol/L, base excess -6.4 mmol/L), and elevated lactate of 4.7 mmol/L (Table 1). The metabolic acidosis was attributed to lactic acidosis secondary to poor tissue perfusion in the context of sustained VT. The hyperglycemia (16.2 mmol/L) was consistent with a stress response. Her initial chest X-ray showed no evidence of pulmonary oedema (Figure 2).

**Table 1:** Venous blood gas on initial presentation pCO₂: partial pressure of carbon dioxide; pO₂: partial pressure of oxygen; ctHb: concentration of total haemoglobin; sO₂: oxygen saturation; FO₂Hb: fraction of oxyhaemoglobin; FCOHb: fraction of carboxyhaemoglobin; FHHb: fraction of deoxyhaemoglobin; FMetHb: fraction of methaemoglobin; cNa⁺: concentration of sodium ions; cK⁺: concentration of potassium ions; cCl⁻: concentration of chloride ions; cCa²⁺: concentration of calcium ions; cGlu: concentration of glucose; cLac: concentration of lactate; ctO₂ac: total oxygen content (arterial); p50c: partial pressure of oxygen at 50% hemoglobin saturation; cBase(Ecf)ic: base excess in extracellular fluid; cHCO₃: standard bicarbonate concentration

Parameter	Result	Unit	Reference range
Blood gas values			
pH	7.267		7.35-7.45
pCO₂	5.99	kPa	4.50-6.00
pO₂	3.2	kPa	10.0-13.0
Oximetry values			
ctHb	144	g/L	115-150
sO₂	31.4	%	
FO₂Hb	31.1	%	
FCOHb	0.6	%	0.5-1.5
FHHb	67.9	%	
FMetHb	0.4	%	0.0-2.0
Electrolyte values			
cNa⁺	128	mmol/L	136-145
cK⁺	3.5	mmol/L	3.5-5.1
cCl⁻	96	mmol/L	98-107
cCa²⁺	1.2	mmol/L	1.15-1.27
Metabolite values			
cGlu	16.2	mmol/L	3.5-5.3
cLac	4.7	mmol/L	0.9-1.7
Temperature-corrected			
pH(T)	7.267		
pCO₂(T)	5.99	kPa	
pO₂(T)	3.2	kPa	
Oxygen status			
ctO₂ac	6.3	Vol%	
p50c	4.32	kPa	
Acid-base status			
cBase(Ecf)ic	-6.4	mmol/L	-2.0 to 3.0
cHCO₃⁻(P.st)ic	17.9	mmol/L	22.0-26.0

**Figure 3:**
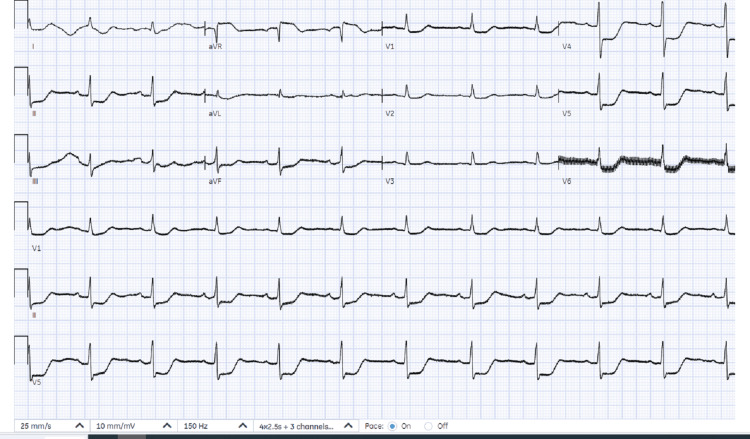
ECG after DCCV Post DCCV ECG showed marked ST-segment depression predominantly affecting lateral leads ECG: electrocardiogram; DCCV: direct current cardioversion

Due to hemodynamic instability, the decision was made to proceed with cardioversion. She was sedated in the emergency department with full resuscitation support, including continuous cardiac and hemodynamic monitoring. Direct current cardioversion (DCCV) was performed with a single 150-joule shock, successfully restoring sinus rhythm (Figure 3), and her blood pressure improved to 145/65 mmHg. The post-cardioversion 12-lead ECG demonstrated widespread ST depression, predominantly in the lateral leads. At this stage, our primary differential diagnosis was ischemia-induced VT, with a corrected QT (QTc) of 412 ms. Subsequently, the emergency cardiology team was consulted for urgent cardiac catheterization. Before catheter laboratory transfer, rhythm stabilization was achieved with intravenous amiodarone: a 300 mg loading dose followed by a 900 mg continuous infusion over 24 hours.

**Figure 4:**
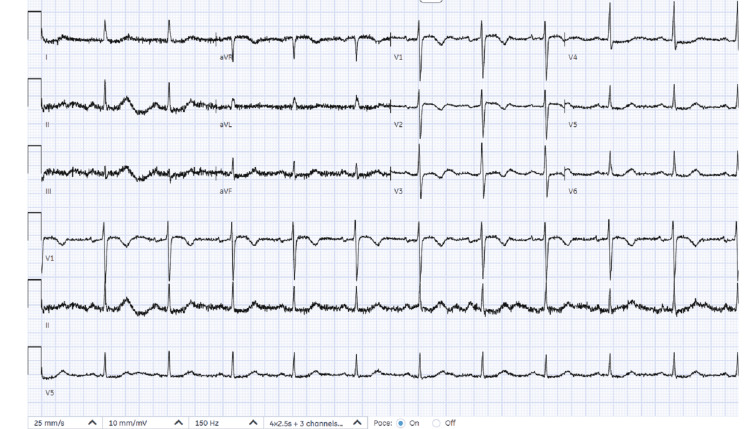
ECG after coronary angiogram This ECG showed anterior T-wave inversions, demonstrating repolarisation abnormality ECG: electrocardiogram

The patient was immediately transferred to the catheterization laboratory and loaded with dual antiplatelet therapy (aspirin 300 mg and ticagrelor 180 mg). Urgent coronary angiography revealed normal coronary arteries, effectively excluding obstructive coronary artery disease as the underlying cause. Following angiography, the post-procedure ECG demonstrated new T-wave inversions in leads V1-V3, suggesting a non-coronary etiology (Figure 4). Transthoracic echocardiography revealed significant structural abnormalities, including thinning and aneurysmal changes of the basal to mid-right ventricular free wall, along with mild concentric left ventricular hypertrophy. Left ventricular ejection fraction was visually estimated at 60-65%. It demonstrated a right ventricular outflow tract (RVOT) measuring 30 mm in the parasternal long-axis (PLAX) view, 34 mm proximally, and 31 mm distally in the parasternal short-axis (PSAX) view. The right ventricular fractional area change (RV FAC) was 28%.

**Figure 5:**
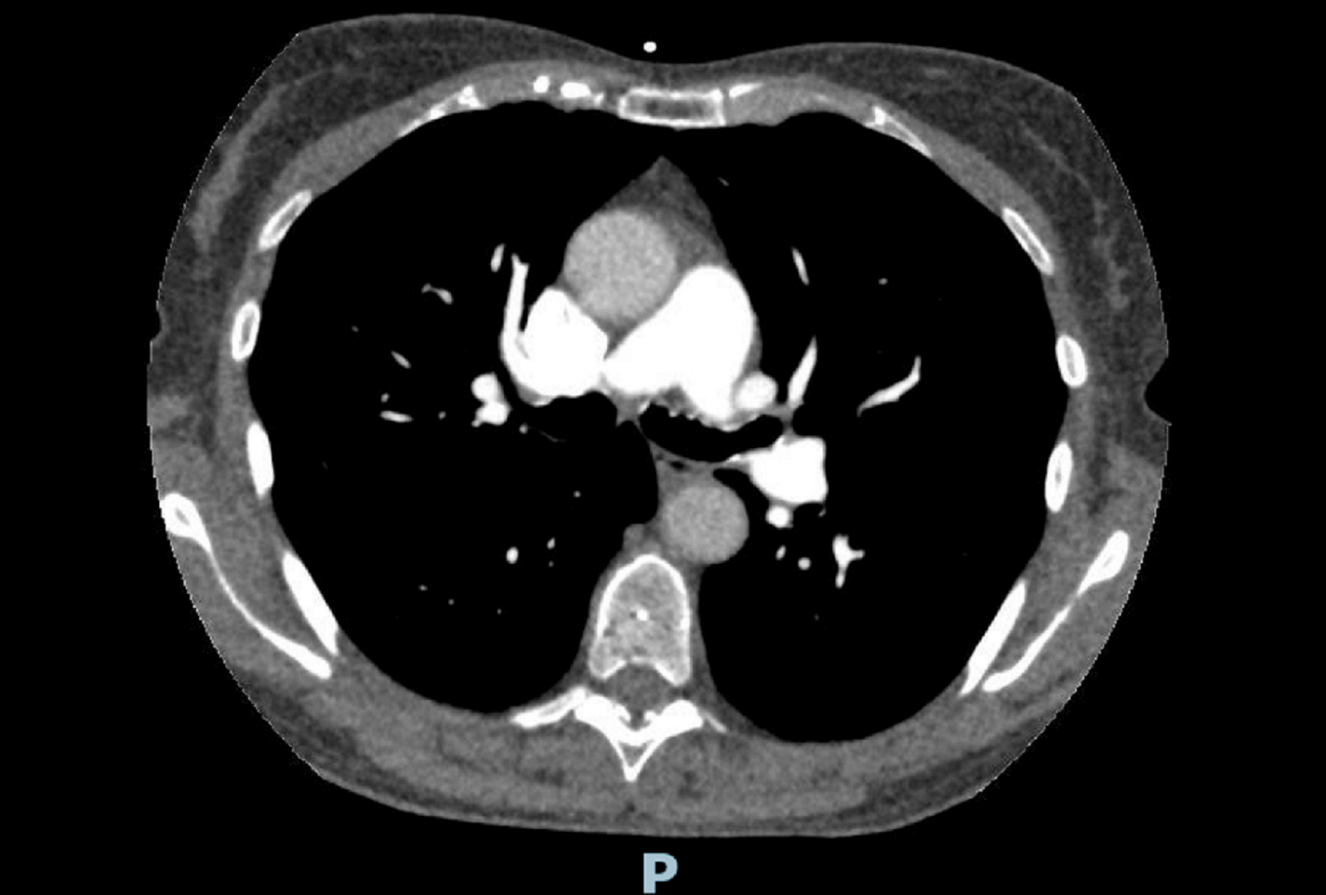
CT pulmonary angiogram (CTPA) CTPA did not show any evidence of pulmonary embolus

Given the elevated D-dimer level of 863 ng/mL, CT pulmonary angiography (CTPA) was performed to exclude pulmonary embolism, which was negative (Figure 5). Serial troponin levels were elevated, peaking at 985 ng/L on day two, despite normal coronary anatomy (Table 2). The persistent mild hyponatremia (129-131 mmol/L) was noted throughout the admission. Cardiac MRI with late gadolinium enhancement identified a 4-cm area of focal bulging and akinesis affecting the basal anterior right ventricular free wall (Video 1). This was associated with late gadolinium enhancement and right ventricular systolic impairment with a right ventricular ejection fraction of 51%. The right ventricle demonstrated a normal end-diastolic volume of 120 mL and EDV/BSA of 81.52 mL/m^2^. The findings were highly suggestive of ARVC, particularly in the context of sustained VT.

**Table 2:** Blood results eGFR: estimated glomerular filtration rate; ALT: alanine aminotransferase

Blood tests	D0	D1	D2	D3	D4	D5	D6	Reference range
Haematocrit (L/L)	0.41	0.36	0.38	0.39	0.39	0.41	0.39	0.36-0.46
Lymphocyte count (× 10^9^/L)	2.8	1.7	1.5	1.7	1.6	1.6	1.5	1.0-3.0
Eosinophils count (× 10^9^/L)	0	0	0	0.1	0	0.1	0.1	0.0-0.5
Neutrophil count (× 10^9^/L)	5.4	3.8	2.6	2.5	2.4	2.9	3.7	2.0-7.0
Haemoglobin estimation (g/L)	136	125	129	132	133	140	130	120-150
Platelet Count (× 10^9^/L)	235	182	185	195	199	228	202	150-410
Mean corpuscular volume (MCV) (fL)	93	92	91	91	91	92	92	83-101
Total white cell count (× 10^9^/L)	8.8	6	4.8	4.9	4.6	5.1	5.9	4.0-10.0
D-dimer level (ng/mL)	863							<500
Serum C-reactive protein level (mg/L)	1	1	1					0-9
Serum troponin T level (ng/L)	43		985	743				<14
Serum Creatinine (µmol/L)	79	64	62	56	58	64	61	45-84
eGFR calculated abbreviated MDRD ( mL/min/1.73m²)	65	84	87	90	89	84	88	-
Serum potassium (mmol/L)	4	3.8	4.1	4.2	4.2	4.3	4.6	3.5-5.0
Serum sodium (mmol/L)	129	130	129	130	130	134	131	132-146
Serum urea level (mmol/L)	4.9	3.4	3.3	2.6	3	3.9	4	2.5-6.7
Serum albumin (g/L)	42	42						35-48
Serum alkaline phosphatase (U/L)	89	81						30-150
Serum ALT level (U/L)		81						0-35
Serum total bilirubin level (µmol/L)		12						0-17
Serum calcium (mmol/L)	2.27							2.20-2.60
Corrected serum calcium level (mmol/L)	2.3							2.2-2.6

**Video 1:** Echocardiography, angiogram, and CMR CMR showing a basal bulge (00:24) CMR: cardiac magnetic resonance

The patient was commenced on sotalol 40 mg three times daily for arrhythmia management. Given her elevated risk for recurrent arrhythmias and sudden cardiac death, a dual-chamber implantable cardioverter-defibrillator (ICD) was implanted for secondary prevention. Exercise restriction was advised, and she was referred to an inherited cardiomyopathy specialist for ongoing care.

Regular follow-up included device monitoring and specialized cardiology review. She has been regularly followed up in the specialist clinic and continues to do well, with no further episodes of ventricular arrhythmia detected on device interrogation. Genetic testing confirmed a pathogenic PKP2 mutation, establishing the definitive diagnosis of ARVC. She is heterozygous for a pathogenic PKP2 truncating variant. Consequently, genetic screening was offered to her son and daughter.

## Discussion

The diagnosis of ARVC presents significant challenges due to its variable clinical presentations and overlap with other cardiac conditions. Our patient initially exhibited symptoms and biomarker elevation characteristic of ACS, including chest pain, ST-segment changes, and elevated troponin levels, leading to initial suspicion of ischemic heart disease. However, normal coronary angiography findings, together with echocardiographic evidence of right ventricular structural abnormalities, prompted further evaluation, which ultimately confirmed ARVC on cardiac MRI. ARVC is frequently misdiagnosed, particularly in elderly patients, as it can mimic conditions including right ventricular outflow tract tachycardia, myocarditis, or Brugada syndrome. In this case, the combination of sustained VT with LBBB morphology and right ventricular structural abnormalities on imaging raised clinical suspicion for ARVC. Subsequent genetic testing revealed a pathogenic PKP2 mutation, one of the most frequently implicated genes in ARVC, providing further diagnostic confirmation.

The Revised Task Force Criteria for ARVC diagnosis incorporate structural, histological, and genetic findings alongside clinical features, including arrhythmias and family history (Table 3) [8]. A definitive diagnosis requires satisfaction of either two major, one major and two minor, or four minor criteria across different categories. Our patient fulfilled three major criteria: right ventricular involvement demonstrated by an RV FAC of 28% and regional wall motion abnormalities with aneurysmal changes on echocardiography, confirmed by cardiac MRI, characteristic ECG repolarization abnormalities with T-wave inversions in precordial leads V1-V3, and identification of a pathogenic PKP2 gene mutation. This constellation of findings established a definitive diagnosis of ARVC.

**Table 3:** Revised Task Force criteria for the diagnosis of ARVC The Revised Task Force Criteria were published in 2010 ARVC: arrhythmogenic right ventricular cardiomyopathy; RV: right ventricle; RVOT: right ventricular outflow tract; PLAX: parasternal long axis view; EDV: end diastolic volume; BSA: body surface area; RVEF: right ventricular ejection fraction; RBBB: right bundle branch block; SAECG: single average electrocardiography; LBBB: left bundle branch block

Category	Major criteria	Minor criteria
I. Imaging	By 2D Echo: regional RV akinesia, dyskinesia, or aneurysm + PLAX RVOT ≥32 mm, PSAX RVOT ≥36 mm, or FAC ≤33%. By CMR: regional RV akinesia, dyskinesia, or dyssynchronous RV contraction + RV EDV/BSA ≥110 ml/m² (male) or ≥100 ml/m² (female) or RVEF ≤40%	By 2D Echo: regional RV akinesia or dyskinesia + PLAX RVOT 29–32 mm, PSAX RVOT 32–36 mm, or FAC 33–40%. By CMR: regional RV akinesia, dyskinesia, or dyssynchronous RV contraction + RV EDV/BSA 100–110 ml/m² (male), 90–100 ml/m² (female), or RVEF 40–45%
II. Endomyocardial biopsy	Residual myocytes ≤60% with fibrous replacement of RV myocardium in ≥1 sample, with or without fatty replacement	Residual myocytes 60–75% with fibrous replacement of RV myocardium in ≥1 sample, with or without fatty replacement
III. Repolarization abnormalities	Inverted T-waves in V1–V3 or beyond in individuals >14 years (without complete RBBB)	Inverted T-waves in V1–V2 in individuals >14 years (without complete RBBB) or in V4, V5, or V6. Inverted T waves in V1–V4 in individuals >14 years (with complete RBBB)
IV. Depolarization/conduction abnormalities	Epsilon wave in right precordial leads (V1–V3)	Late potentials on SAECG (≥1 of 3 parameters: fQRS ≥114 ms, low-amplitude signal ≥38 ms, RMS voltage ≤20 μV). Terminal QRS activation ≥55 ms in V1–V3 (without RBBB)
V. Arrhythmias	Sustained or non-sustained VT of LBBB morphology with superior axis	Non-sustained or sustained VT of RV outflow configuration (LBBB morphology with inferior axis) or ≥500 ventricular extrasystoles per 24 hours
VI. Family history	ARVC confirmed in a first-degree relative or pathogenic gene mutation associated with ARVC	ARVC history in a first-degree relative without a definitive diagnosis. Premature sudden death (<35 years) suspected of ARVC in a first-degree relative

In ARVC, fibrofatty replacement of myocardium disrupts electrical conduction by impairing cell-to-cell coupling, reducing connexin-43 expression, and slowing conduction through decreased Nav1.5 channels [9]. These changes create reentrant circuits predisposing to arrhythmias. Ischemic-like ECG changes result from microvascular dysfunction, regional wall motion abnormalities, and altered repolarization patterns. ST depression occurs due to differential repolarization, subendocardial involvement, and ventricular dilation [10-11]. When ARVC extends to the left ventricle, it can produce ischemic-appearing patterns, indicating advanced disease with increased arrhythmic risk.

The management of ARVC focuses primarily on preventing sudden cardiac death, particularly in patients with ventricular arrhythmias. ICDs represent the cornerstone of therapy for high-risk patients. In this case, given the patient's presentation with sustained VT requiring cardioversion, secondary prevention with ICD implantation was appropriately indicated. Additional therapeutic options include catheter ablation for refractory arrhythmias and heart transplantation for advanced heart failure or recurrent ventricular arrhythmias unresponsive to medical therapy. Antiarrhythmic medications, such as the sotalol prescribed in this case, provide adjunctive rhythm control.

Exercise restriction constitutes a critical management component, particularly for patients with PKP2 mutations, as physical exertion increases sudden death risk [12]. Patients with ARVC and ICDs should avoid high-intensity or competitive sports due to increased ventricular arrhythmia risk. Low-intensity activities such as walking, yoga, and recreational cycling are generally permissible at moderate levels, with attention to adequate hydration and avoiding excessive exertion. Exercise recommendations require individualization based on disease severity and regular cardiology follow-up for monitoring and adjustment. Our patient received initial advice to restrict intense physical activity during her recovery phase and was referred to the local inherited cardiomyopathy specialist network for ongoing management.

Genetic counseling and family screening are essential given ARVC's heritable nature. Early identification of at-risk relatives enables timely intervention and management. Following identification of the pathogenic PKP2 variant, our patient's children were offered genetic testing.

## Conclusions

This report highlights the importance of considering ARVC in the differential diagnosis of patients presenting with chest pain, elevated troponin levels, and normal coronary angiography. While ARVC typically presents in younger individuals, this case demonstrates that a substantial minority of patients can present at older ages, particularly with sustained VT, underscoring the need for heightened clinical suspicion in elderly patients with unexplained ventricular arrhythmias. Late-presenting ARVC patients carry significant arrhythmic risk comparable to those with early-onset disease, making ICD therapy a crucial consideration for preventing sudden cardiac death. The diagnostic challenge in elderly patients often leads to initial misdiagnosis, as demonstrated by this patient's initial presentation mimicking ACS. This report underscores the critical importance of comprehensive cardiac imaging, genetic testing, and systematic application of the Revised Task Force Criteria in establishing an accurate diagnosis and guiding appropriate management. Early recognition and proper risk stratification are essential for optimizing outcomes in this challenging patient population.
